# Association between visceral fat and influenza infection in Japanese adults: A population-based cross-sectional study

**DOI:** 10.1371/journal.pone.0272059

**Published:** 2022-07-26

**Authors:** Keita Kinoshita, Naoki Ozato, Tohru Yamaguchi, Kenta Mori, Yoshihisa Katsuragi, Takuji Yasukawa, Koichi Murashita, Shigeyuki Nakaji, Kazushige Ihara

**Affiliations:** 1 Department of Active Life Promotion Sciences, Graduate School of Medicine, Hirosaki University, Aomori, Japan; 2 Health & Wellness Products Research Laboratories, Kao Corporation, Tokyo, Japan; 3 Department of Social Medicine, Graduate School of Medicine, Hirosaki University, Aomori, Japan; 4 Department of Preemptive Medicine, Innovation Center of Health Promotion, Graduate School of Medicine, Hirosaki University, Hirosaki, Japan; 5 COI Research Initiatives Organization, Graduate School of Medicine, Hirosaki University, Aomori, Japan; University of Hawai’i at Manoa College of Tropical Agriculture and Human Resources, UNITED STATES

## Abstract

**Background:**

Several studies have reported that obesity is associated with influenza infection; however, the role of visceral fat remains unclear. The aim of this study was to investigate the association between visceral fat and influenza infection in community-dwelling Japanese adults.

**Methods:**

A cross-sectional study was performed using data from an annual community-based health check-up conducted from May to June in 2019. In total, 1,040 Japanese adults aged 20–89 years were enrolled in this study. Influenza infection status was determined by participants’ responses to a self-administered questionnaire. The visceral fat area (VFA) was measured using a bioimpedance-type visceral fat meter. Participants were classified into four groups using the following cut-off points: VFA < 100 cm^2^ was set as the reference category according to the Japanese criteria, 100 ≤ VFA < 150 cm^2^, 150 ≤ VFA < 200 cm^2^, and 200 cm^2^ ≤ VFA. Logistic regression models were used to assess the association between VFA and influenza infection.

**Results:**

In total, 119 participants had influenza infections in the past year. In the multivariate adjusted model, a higher VFA was significantly associated with increased influenza infection; the adjusted odds ratio for 200 cm^2^ ≤ VFA was 5.03 [95% confidence interval (CI): 1.07–23.6], that for 150 ≤ VFA < 200 cm^2^ was 1.97 (95% CI: 0.71–5.45), and that for 100 ≤ VFA < 150 cm^2^ was 1.62 (95% CI: 0.84–3.12), compared with that for VFA < 100 cm^2^ (*p* for trend = 0.049). These findings were confirmed in the same cohort the following year.

**Conclusions:**

Our results suggest that visceral fat accumulation is associated with influenza infection. Large-scale prospective studies using diagnostic information for influenza infection are required to confirm this association.

## Introduction

Seasonal influenza is one of the most serious infectious diseases worldwide and is estimated to result in up to 5 million cases of severe illness and up to 650,000 respiratory-related deaths per year [1]. In Japan, approximately 10 million people are infected with influenza, and 10,000 people are estimated to die due to influenza annually [2]. Although several recommendations, such as hand washing and vaccination, have been made, they are insufficient for influenza prevention.

Metabolic disorders have been widely recognized as one of the risk factors for seasonal influenza in adults [3–6]. Obesity has been recognized as a risk factor for increased influenza severity since the novel influenza A virus strain (H1N1) was discovered [3, 4]. Obesity is associated with dysregulation of the immune system, which leads to the development of autoimmune disease and impairment of protective immunity [5–7]. Specifically, the immune response to influenza vaccination is impaired by obesity [8]. Several epidemiological studies have reported that general obesity, defined by body mass index (BMI), is independently associated with influenza infection [9–12]. However, most of these studies included Westerners and not Asian populations.

BMI is frequently used in clinical settings to assess obesity status; however, it reflects fat content and muscle mass [13]. In addition, BMI cannot distinguish visceral fat from subcutaneous fat [14]. Recent studies have shown that abdominal obesity, defined by visceral fat area (VFA), has stronger associations with metabolic disorders (such as hypertension, type 2 diabetes, and dyslipidemia) than with BMI [15–17]. Furthermore, Asian individuals have a higher susceptibility to these diseases, with visceral fat accumulation noted even in individuals with a lower BMI, compared with Westerners [18]. Therefore, reducing the VFA might be more effective than reducing the BMI, in decreasing the risk of influenza in Asian populations. However, whether influenza severity or infection is associated with visceral fat accumulation in adults remains unclear not only in Asian population but also in Westerners.

This study aimed to examine the association between VFA, as well as BMI, and influenza infection in community-dwelling Japanese adults. This was achieved by analyzing data from two health check-ups separately to confirm the reproducibility of this association.

## Materials and methods

### Participants

The Iwaki Health Promotion Project, launched in 2005, is an annual health check-up. The participants were adult men and women living in the Iwaki region of Hirosaki city, Aomori prefecture, Japan [19]. All adult residents (approximately 6,000 people aged ≥20 years as of 2019) in this region were invited based on their resident registration. Approximately 10% of these adult residents participated voluntarily in the annual health check-up. Therefore, the inclusion criterion of this study was all adults (aged ≥20 years) who attended the health check-up. The exclusion criterion was participants who did not provide written informed consent. The present analysis was performed using data obtained from the 2019 and 2020 health check-ups, as a population-based cross-sectional study. The study was approved by the Ethics Committee of Hirosaki University School of Medicine (2019–009 and 2020-046-1) and conducted in accordance with the principles of the Declaration of Helsinki. Written informed consent was obtained from all the participants before each health check-up. This study was registered in the University Hospital Medical Information Network (UMIN-CTR, https://www.umin.ac.jp) prior to the analysis (UMIN ID: UMIN000036741).

### Influenza infection

Influenza infection was assessed using a self-administered questionnaire according to a previous study [20]. The participants were asked if they had been infected with influenza in the past year (yes/no). They were expected to answer the questionnaire based on the doctor’s diagnosis using a commercial rapid diagnostic test kit, which has high sensitivity (97.1%) and specificity (89.2%) and is commonly used in clinical settings, such as outpatient clinics, in Japan [21, 22].

### Visceral fat measurement

VFA was measured using a bioimpedance-type visceral fat meter (EW-FA90; Panasonic Corporation, Osaka, Japan), which is a certified medical device in Japan (No. 22500BZX00522000) for measuring VFA non-invasively [23]. Measurements obtained using this device were reported to be strongly correlated with those obtained using computed tomography, which is the gold standard for VFA measurement [24].

According to the Japanese criteria for “obesity disease,” VFA < 100 cm^2^ was set as the reference category [25]. In addition, we set the cut-off points as follows: 100 ≤ VFA < 150 cm^2^, 150 ≤ VFA < 200 cm^2^, and 200 cm^2^ ≤ VFA.

### Measurement of other parameters

Data on smoking habits (% current), alcohol intake (% current), exercise habit (% yes), number of habitual medications (diabetes, hypertension, and dyslipidemia), number of individuals living in the household (hereafter referred to as household size), education, and self-rated health scores were obtained through self-administered questionnaires. The self-rated health score was five-grade evaluation assessed by the question, “In general, would you say your health is (excellent, very good, good, fair, or poor).” The following clinical parameters were also measured: height; weight; BMI (calculated from height and body weight); systolic blood pressure; diastolic blood pressure; and blood glucose, hemoglobin A1c (HbA1c), low-density lipoprotein cholesterol (LDL cholesterol), high-density lipoprotein cholesterol (HDL cholesterol), and serum triglyceride levels. Blood samples were collected from the peripheral veins after ≥ 9 h fasting state. All laboratory measurements were outsourced to LSI Medience Co. (Tokyo, Japan) and conducted according to their standard operating procedure. BMI < 25 kg/m^2^ was set as the reference, and the cut-off points were 25 ≤ BMI ≤ 30 kg/m^2^ (obese class 1), 30 ≤ BMI < 35 kg/m^2^ (obese class 2), and 35 kg/m^2^ ≤ BMI (obese class 3 and 4) [25, 26]. Hypertension was defined as blood pressure ≥ 140/90 mmHg or the use of antihypertensive drugs [27]. Diabetes was defined as fasting blood glucose level ≥ 126 mg/dL, HbA1c  level ≥ 6.5%, or the use of antidiabetic drugs [28]. Dyslipidemia was defined as LDL cholesterol level  ≥ 140 mg/dL, HDL cholesterol  level < 40 mg/dL, triglyceride level ≥ 150 mg/dL, or the use of antidyslipidemic drugs [29].

### Statistical analysis

Participant characteristics are reported as mean ± standard deviation (SD) or percentage. The Mann–Whitney U test and Fisher’s exact test were used for comparisons between two groups. Logistic regression analysis was performed to investigate odds ratio (OR) and 95% confidence interval (CI) for influenza infection, comparing participants in the higher VFA/BMI groups to those in the lowest group. Tests for linear trends across the VFA/BMI groups were conducted by assigning ordinal numbers to the increasing category of the VFA/BMI group. As for the confirmatory study, only the tests for linear trends were conducted because of the small sample size in the highest VFA/BMI group.

Statistical tests were two-tailed, and statistical significance was set at *p* < 0.05. All analyses were performed using SPSS version 25 (SPSS Inc., Chicago, IL, USA).

## Results

In 2019, 1,065 individuals participated in the health check-up from May 25 to June 3. Of these individuals, 25 did not complete the questionnaire or clinical assessments and were excluded from the analysis. Finally, 1,040 participants were included in the analysis. Of them, 617 (59.3%) were women, and the mean (SD) age was 52.5 (15.1) years. Among the participants, 119 individuals experienced influenza infection in the past year. Table 1 shows the characteristics of the participants classified by influenza infection status (yes/no). Participants who experienced influenza infection in the past year were younger (*p* = 0.024) and had larger household sizes (*p* = 0.036), and lower proportion of hypertension (*p* = 0.042). No other significant differences were observed between the groups.

**Table 1:** Participant characteristics classified based on experience of influenza infection in the past year.

	Experience of influenza infection in the past year		
	No (n = 921)	Yes (n = 119)	*p*-value
Age (year)	52.8 (15.1)	49.3 (15.4)	0.024
Woman (%)	58.6	64.7	0.234
Smoking status (% current)	17.5	14.3	0.439
Alcohol intake (% current)	8.8	13.4	0.129
Exercise habits (% yes)	22.1	22.7	0.907
Self-rated health score	3.1 (0.2)	3.1 (0.7)	0.880
Number of habitual medications	0.4 (0.7)	0.3 (0.6)	0.177
Household size (number of individuals)	3.8 (1.6)	4.2 (1.9)	0.036
Education			
<9 years	10.7	6.7	0.104
9–12 years	54.8	51.3	
≥12 years	34.0	40.3	
Others	0.4	1.7	
Visceral fat area (cm^2^)	81.3 (46.0)	77.9 (48.7)	0.291
Body mass index (kg/m^2^)	23.0 (3.6)	22.7 (3.6)	0.251
Hypertension (% yes)	36.5	26.9	0.042
Diabetes (% yes)	8.4	12.6	0.125
Dyslipidemia (% yes)	38.4	35.3	0.548

Values are presented as mean (SD) or percentages. The Mann–Whitney U test was used for continuous variables, and Fisher’s exact test was used for categorical variables.

Table 2 shows the OR (95% CI) for influenza infection according to the VFA group among 2019 participants. After multivariate adjustment, the higher VFA group was significantly associated with increased influenza infection (Model 2: *p* for trend = 0.049). The OR for the VFA ≥ 200 cm^2^ group was 5.03 (95% CI: 1.07–23.6, *p* = 0.041), that for the 150 cm^2^ ≤ VFA < 200 cm^2^ group was 1.97 (95% CI: 0.71–5.45), and that for the 100 cm^2^ ≤ VFA < 150 cm^2^ group was 1.62 (95% CI: 0.84–3.12), compared with that for the VFA < 100 cm^2^ group.

**Table 2:** Association of visceral fat area (VFA) with influenza infection according to the VFA group in 2019.

	VFA				
	VFA<100 cm^2^	100 ≤ VFA < 150 cm^2^	150 ≤ VFA < 200 cm^2^	200 cm^2^ ≤ VFA	*p* for trend
Experience of influenza infection in the past year (yes/no)	84/648	23/188	8/67	4/18	
Crude	1.00 (ref.)	0.94 (0.58, 1.54)	0.92 (0.43, 1.99)	1.71 (0.57, 5.19)	0.771
Model 1	1.00 (ref.)	1.61 (0.85, 3.05)	2.00 (0.75, 5.33)	4.96 (1.12, 22.0)*	0.040
Model 2	1.00 (ref.)	1.62 (0.84, 3.12)	1.97 (0.71, 5.45)	5.03 (1.07, 23.6) *	0.049

Values presented are odds ratios (95% confidence intervals). Logistic regression analyses were performed. Model 1 was adjusted for age, sex, and BMI. Model 2 was adjusted for Model 1 plus smoking habits, alcohol intake, exercise habits, self-rated health score, household size, education, hypertension, diabetes, and dyslipidemia.

* p < 0.05

Table 3 shows the OR (95% CI) for influenza infection according to the BMI group. There was no significant association between BMI and influenza infection (model 2: *p* for trend = 0.356).

**Table 3:** Association of body mass index (BMI) with influenza infection according to the BMI group in 2019.

	BMI				
	BMI <25 kg/m^2^	25 ≤ BMI < 30 kg/m^2^	30 ≤ BMI < 35 kg/m^2^	35 kg/m^2^ ≤ BMI	*p* for trend
Experience of influenza infection in the past year (Yes/No)	91/683	24/199	3/29	1/10	
Crude	1.00 (ref.)	0.91 (0.56, 1.46)	0.78 (0.23, 2.0)	0.75 (0.09, 5.93)	0.545
Model 1	1.00 (ref.)	0.84 (0.46, 1.55)	0.55 (0.13, 2.29)	0.44 (0.04, 4.41)	0.354
Model 2	1.00 (ref.)	0.84 (0.45, 1.56)	0.54 (0.13, 2.32)	0.44 (0.04, 4.62)	0.356

Values are adjusted odds ratios (95% confidence intervals). Logistic regression analyses were performed. Model 1 was adjusted for age, sex, and VFA. Model 2 was adjusted for Model 2 plus smoking habits, alcohol intake, exercise habits, self-rated health score, household size, education, hypertension, diabetes, and dyslipidemia.

To validate the reproducibility of the association between VFA or BMI and influenza infection, we analyzed the data obtained in the 2020 health check-up from September 17 to September 25. The number of participants in the 2020 check-up was approximately half of that in a typical year because of the COVID-19 pandemic. In 2020, 524 individuals participated in the health check-up, and among them, two did not have complete VFA measurement details. In total, 522 individuals (82% of whom also participated in the 2019 health check-up) who participated in the 2020 health check-up were enrolled in a confirmatory study. The characteristics of the confirmation group are presented in S1 Table. The results of the analysis on VFA or BMI and influenza infection in the confirmatory study were similar to those of the main study (S2 and S3 Tables); higher VFA was significantly associated with influenza infection (Model 2: *p* for trend = 0.002), whereas no significant association was observed between BMI and influenza infection (Model 2: *p* for trend = 0.741).

## Discussion

To the best of our knowledge, this is the first study to investigate the association between visceral fat and influenza infection. We found that a higher VFA was significantly associated with increased rate of influenza infection in community-dwelling Japanese adults. This result is consistent with limited epidemiological findings for the association between abdominal obesity an influenza infection. In Korean school-aged children, a higher waist circumference was significantly associated with increased H1N1 influenza infection [30]. These present and previous studies support the hypothesis that visceral fat increases the risk of influenza infection.

Although little has been reported on the association between VFA and influenza, there is increasing evidence regarding the association between visceral fat and COVID-19, which spread in a similar way and is one of the most severe respiratory diseases. A recent systematic review and meta-analysis reported that visceral fat is associated with COVID-19 severity [31, 32]. This could be attributed to the malfunction of visceral fat, which impairs the immune system by secreting inflammatory adipokines [33]. In addition, adiponectin, a unique adipokine, has been shown to exhibit anti-inflammatory effects on other cell types, such as macrophages and fibrogenic cells. A previous study demonstrated that visceral fat was significantly negatively associated with adiponectin, whereas the association with BMI was not [34], which may have led to the increased influenza infection incidence in higher VFA group compared to other groups in this study. In contrast, muscles exhibit an anti-inflammatory activity [35]. Interleukin‐15 (IL-15), one of the skeletal muscle-derived cytokines, is required for the development and survival of natural killer lymphocytes. IL-15 also has important effects on adipose tissue, such as inhibiting adipocyte differentiation. Poros et al. [36] reported that decreased muscle mass is associated with COVID-19 mortality, which is in line with this potential mechanism for the association between visceral fat or muscle mass and COVID-19 severity. It is necessary to consider not only visceral fat but also muscle mass to assess the outcome of the infection.

In this study, we did not find a significant association between BMI and influenza infection. Several studies have demonstrated that BMI is significantly associated with influenza infection or severity [9–12]. One possible explanation for this discrepancy is the difference in the study sample races. Asian individuals have a higher susceptibility to visceral fat accumulation, even in individuals with a lower BMI, than Westerners [18, 37]. Specifically, the classifications of general obesity for Japanese and Westerners are BMI ≥ 25 kg/m^2^ and BMI ≥ 30 kg/m^2^, respectively, whereas those for abdominal obesity are comparable. The number of our study participants whose BMI was ≥30 kg/m^2^, the cut-off point of which reported a significantly higher risk for influenza infection than the control (22.5 < BMI ≤ 25 kg/m^2^) [10], was very small (approximately 4% of the total sample). This may be because our study participants were relatively healthy community-dwelling people. Overall, abdominal obesity defined by visceral fat is a more useful indicator of obesity and related infectious diseases than general obesity defined by BMI in Asian populations.

The strengths of this study include a wide age range with VFA measurements obtained using an abdominal bioimpedance method. However, this study had some limitations. First, given its cross-sectional design, a strong causal relationship could not be established. Second, influenza infection status was determined using a self-administered questionnaire, which can cause recording bias and misclassification of other bacterial infections. Moreover, the determination of influenza infection using questionnaires may be limited to countries with high-quality and easy-access medical care, such as in Japan. Third, we could not collect vaccine-related information, which is strongly associated with influenza infection status. In Japan, the influenza vaccination rate has been approximately 40% in recent years [38]. A previous meta-analysis showed that adults with obesity are more likely to receive influenza vaccination [39], suggesting an underestimation of the association between visceral fat and influenza infection. Finally, the number of our study participants with BMI ≥ 30 kg/m^2^ was small; therefore, our findings regarding the association between BMI and influenza should be interpreted with caution. Further studies including a more significant number of adults with obesity are required in Asian populations to evaluate the association of BMI with influenza infection.

## Conclusions

Our results suggest that visceral fat accumulation is associated with influenza infection in Japanese adults. These data could aid in preventing infectious diseases by raising awareness regarding the importance of the management of abdominal obesity. Further large-scale prospective studies are required to confirm this association.

## Supporting information

S1 Table Participant characteristics classified based on influenza infection in 2020 health check-up. Values are presented as mean (SD) or percentages. The Mann–Whitney U test was used for variables, and Fisher’s exact test was used for categorical variables.
(DOCX)

S2 Table Association of visceral fat area with influenza infection according to the VFA group in 2020. Logistic regression analyses were performed. Model 1 was adjusted for age, sex, and BMI Model 2 was adjusted for Model 1 plus smoking habits, alcohol intake, exercise habits, self-rated health score, household size, education, hypertension, diabetes, and dyslipidemia.
(DOCX)

S3 Table Association of body mass index with influenza infection according to the BMI group in 2020. Logistic regression analyses were performed. Model 1 was adjusted for age sex, and VFA. Model 2 was adjusted for Model 1 plus smoking habits, alcohol intake, exercise habits, self-rated health score, household size, education, hypertension, diabetes, and dyslipidemia.
(DOCX)
