# Supplementary material for: Association between visceral fat and influenza infection in Japanese adults: A population-based cross-sectional study
Source: PLoS One. 2022 Jul 26;17(7):e0272059. doi: 10.1371/journal.pone.0272059 (PMC9321422; doi:10.1371/journal.pone.0272059)
Supplement: S1 Table — Values are presented as mean (SD) or percentages. The Mann–Whitney U test was used for variables, and Fisher’s exact test was used for categorical variables. (DOCX) [file pone.0272059.s001.docx]

**S1 Table. Participant characteristics classified based on influenza infection in 2020 health check-up.**

|  | Experience of influenza infection in the past year | |  |
| --- | --- | --- | --- |
|  | No (n = 490) | Yes (n = 32) | *p*-value |
| Age (year) | 51.3 (15.3) | 52.5 (15.9) | 0.841 |
| Woman (%) | 59.0 | 71.9 | 0.193 |
| Smoking status (% current) | 18.4 | 6.3 | 0.095 |
| Alcohol intake (% current) | 47.1 | 56.3 | 0.363 |
| Exercise habits (% yes) | 35.9 | 40.6 | 0.576 |
| Self-rated health score | 3.0 (0.7) | 3.2 (0.7) | 0.880 |
| Number of habitual medications | 0.3 (0.6) | 0.5 (0.9) | 0.177 |
| Household size | 3.7 (1.8) | 4.0 (1.8) | 0.314 |
| Education | | | |
| <9 years | 6.9 | 9.4 | 0.790 |
| 9–11 years | 53.1 | 50.0 |  |
| $\geq12 years$ | 39.4 | 40.6 |  |
| Others | 0.6 | 0.0 |  |
| Visceral fat area (cm^2^) | 83.0 (44.0) | 84.1 (54.7) | 0.834 |
| Body mass index (kg/m^2^) | 22.8 (3.3) | 22.7 (4.2) | 0.850 |
| Hypertension (% yes) | 39.2 | 43.8 | 0.709 |
| Diabetes (% yes) | 8.4 | 9.4 | 0.745 |
| Dyslipidemia (% yes) | 44.5 | 53.1 | 0.364 |

Values are presented as mean (SD) or percentages. The Mann-Whitney U test was used for continuos variables, and Fisher's exact test was used for categorical variables.
