# Supplementary material for: Association between visceral fat and influenza infection in Japanese adults: A population-based cross-sectional study
Source: PLoS One. 2022 Jul 26;17(7):e0272059. doi: 10.1371/journal.pone.0272059 (PMC9321422; doi:10.1371/journal.pone.0272059)
Supplement: S2 Table — Logistic regression analyses were performed. Model 1 was adjusted for age, sex, and BMI Model 2 was adjusted for Model 1 plus smoking habits, alcohol intake, exercise habits, self-rated health score, household size, education, hypertension, diabetes, and dyslipidemia. (DOCX) [file pone.0272059.s002.docx]

**S2 Table. Association of visceral fat area (VFA) with influenza infection** **according to the VFA group in 2020.**

|  | VFA | | | | *p* for trend | | |
| --- | --- | --- | --- | --- | --- | --- | --- |
|  | VFA<100 cm^2^ | 100 ≤ VFA < 150 cm^2^ | 150 ≤ VFA < 200 cm^2^ | 200 cm^2^ ≤ VFA | Crude | Model 1 | Model 2 |
| Experience of influenza infection in the past year (yes/no) | 19/336 | 8/115 | 4/34 | 1/5 | 0.128 | 0.001 | 0.002 |

Logistic regression analyses were performed. Model 1 was adjusted for age, sex, and BMI. Model 2 was adjusted for Model 1 plus smoking habits, alcohol intake, exercise habits, self-rated health score, household size, education, hypertension, diabetes, and dyslipidemia.
