# Supplementary material for: Association between visceral fat and influenza infection in Japanese adults: A population-based cross-sectional study
Source: PLoS One. 2022 Jul 26;17(7):e0272059. doi: 10.1371/journal.pone.0272059 (PMC9321422; doi:10.1371/journal.pone.0272059)
Supplement: S3 Table — Logistic regression analyses were performed. Model 1 was adjusted for age sex, and VFA. Model 2 was adjusted for Model 1 plus smoking habits, alcohol intake, exercise habits, self-rated health score, household size, education, hypertension, diabetes, and dyslipidemia. (DOCX) [file pone.0272059.s003.docx]

**S3 Table. Association of body mass index (BMI) with influenza infection** **according to the BMI group in 2020.**

|  | BMI | | | | *p* for trend | | |
| --- | --- | --- | --- | --- | --- | --- | --- |
|  | BMI <25 kg/m^2^ | 25 ≤ BMI < 30 kg/m^2^ | 30 ≤ BMI < 35 kg/m^2^ | 35 kg/m^2^ ≤ BMI | Crude | Model 1 | Model 2 |
| Experience of influenza infection in the past year (yes/no) | 23/370 | 8/109 | 1/10 | 0/2 | 0.659 | 0.830 | 0.741 |

Logistic regression analyses were performed. Model 1 was adjusted for age, sex, and VFA. Model 2 was adjusted for Model 1 plus smoking habits, alcohol intake, exercise habits, self-rated health score, household size, education, hypertension, diabetes, and dyslipidemia.
